# Effects of Initial Experiences on Risky Choice

**DOI:** 10.1177/17470218261432610

**Published:** 2026-03-04

**Authors:** Elliot A. Ludvig, Neil McMillan, Jeffrey M. Pisklak, Nick Simonsen, Alice Mason, Jason Long, Marcia L. Spetch, Christopher R. Madan

**Affiliations:** 1 Department of Psychology, University of Warwick, Coventry, UK; 2 Department of Psychology, University of Alberta, Edmonton, AB, Canada; 3 Medical School, University of Adelaide, SA, Australia; 4 Department of Management, Aarhus University, Denmark; 5 School of Psychology, University of Nottingham, UK; 6 Department of Psychology, University of Bath, England, UK

**Keywords:** risky choice, primacy effects, relative value, extreme-outcome effect, decisions from experience, gambling

## Abstract

When people make risky choices based on prior experience, biases in learning and memory can affect their preferences. One such bias is the primacy effect, whereby outcomes experienced during initial learning disproportionately affect later memory and choice. Here, we investigated potential primacy effects with two features of risky options: outcome probability and relative value. In the first two experiments (total *N* = 382), the order of experiencing different outcome probabilities was varied across three groups, and neither experiment revealed any primacy effects. In the last experiment (total *N* = 390), the relative value of outcomes was manipulated by including an extra wildcard option. This wildcard option sometimes had more extreme outcomes than the rest of the choice set and sometimes had more moderate outcomes. The order of experiencing the more-extreme wildcard option was manipulated to evaluate potential primacy effects. During initial learning, including the more-extreme wildcard option affected choice as compared to a group with a wildcard that yielded moderate outcomes; adding that same more-extreme wildcard later in the session, however, had no effect on choice. Together, these results suggest that risk preferences based on relative value show a lasting primacy effect, but that learning about outcome probabilities is more continuously updated.

## Introduction

Life is full of choices that involve risk. Should you return to your favorite vacation spot or try a new one? Should you put your money in guaranteed savings or risk it on stocks? Should you quit gambling after a win or keep playing? As famously shown by Kahneman and Tversky (1979), people’s tendency to choose riskier options is not based exclusively on the objective expected values; instead, people show systematic biases depending on factors such as the probabilities, how the choices are framed, and the relative values of outcomes. Moreover, these biases differ, and are sometimes reversed, depending on whether information about the probabilities and outcomes is described or learned through personal experience (Hertwig & Erev, 2009; Olschewski et al., 2024). For example, people tend to overweight rare events when deciding based on described probabilities, but they underweight rare events when choosing based on experience (Hertwig et al., 2004). People are more risk-seeking for losses than for gains when deciding based on description but can show the opposite tendency when choosing based on experience (Ludvig & Spetch, 2011; Madan et al., 2017).

**Figure 1.**
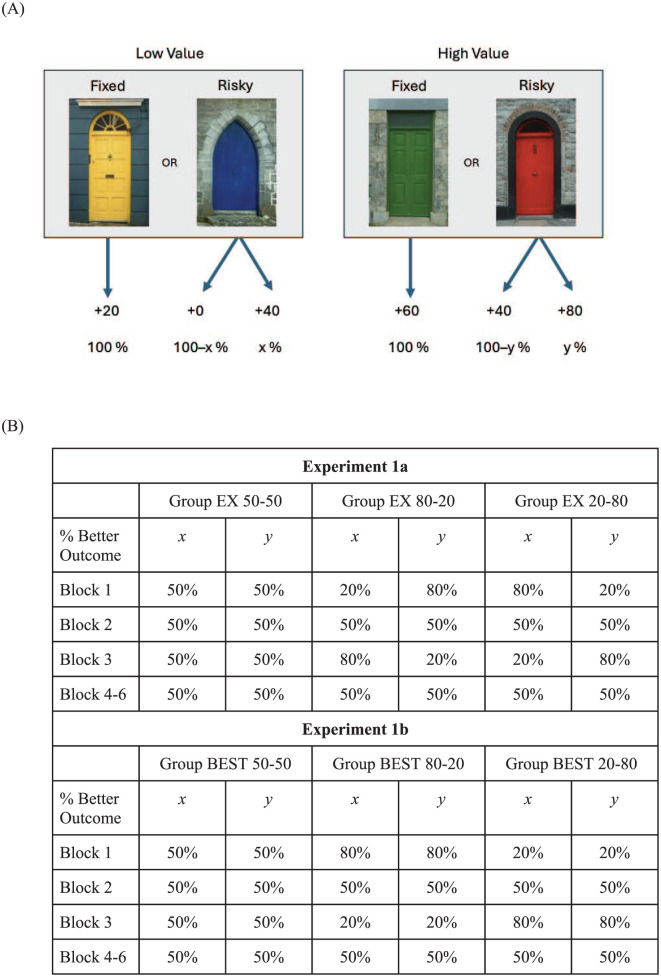
Procedure for the basic risky-choice task. (A) Schematic of the task. People chose between pairs of doors. Each fixed door provided the same number of points whenever it was selected, whereas the risky doors provided a lower or higher number of points. The probabilities of receiving either of the two possible outcomes on the risky doors (*x* and *y*) were manipulated across blocks in Experiments 1a and 1b. In Experiment 2, all risky outcomes occurred with a 50/50 chance throughout the experiment, but a third risky option (wildcard) was included, and the outcome values for the wildcard varied across blocks. (B) Table of outcome values for each group and block in Experiments 1a and 1b. For the risky options, the experiment systematically manipulated the percentage of the time that the better outcome was delivered, as denoted by the variations in *x* and *y* values across blocks.

For decisions based solely on experience, people need to learn about the options through feedback from their choices. Consider a scenario in which people are given repeated choices between pairs of options, as shown in Figure 1. Feedback (points earned) can be used to learn the expected value of each option, and most people readily learn to choose the higher-value options over the lower-value options. Feedback can also be used to learn the outcome probabilities. In this example, within each value set, one option always (100% chance) gives the same magnitude win, whereas the other option is risky with a chance of a larger or smaller win.

Given that decisions from experience rely on sequential learning, the order of experience with wins and losses could potentially affect risky choices. For example, the most recent outcomes experienced on an option disproportionately affect experience-based risky choice (see Wulff et al., 2018, for a review). This recency bias may reflect a greater tendency for more recent experiences to be sampled in memory (e.g., Hertwig et al., 2004; Plonsky & Erev, 2017; Plonsky et al., 2015). In the domain of gambling, recent strings of wins or losses both affect betting behavior (e.g., Abe et al., 2021). For example, strings of wins may increase risky choice by triggering a “winning-streak” or “hot-hand” belief, and strings of losses may increase risky choice by triggering the “gambler’s fallacy” (i.e., the false belief that a win is overdue and more likely to occur). Both types of recency biases have been demonstrated in lab-based tasks, as well as in real-world gambling situations such as casino roulette (Croson & Sundali, 2005; Sundali & Croson, 2006; Xu & Harvey, 2014).

Risk preferences may also be subject to primacy effects in which the initial outcomes experienced have a disproportional and persistent effect on choice. Within the gambling literature, some survey research has identified experience with a big win early in gambling as a factor in problem gambling (e.g., Custer, 1984; Turner et al., 2006), although experimental work has sometimes failed to provide supporting evidence for this effect (e.g., Rune et al., 2012; St-Amand et al., 2018; Weatherly et al., 2004). An analysis of a large set of repeated risky-choice trials in an experimental operant-learning task (Shteingart et al., 2013), however, showed that the outcome of the very first experience with a risky option had a lasting effect on people’s choices (see also Madan, 2024; Sinclair et al., 2024). The initial outcomes experienced also have relatively long-lasting effects in tasks involving stock-market decisions (Lejarraga et al., 2016), sports betting (Edson et al., 2021), predicting coin tosses (Eben et al., 2023; Langer & Roth, 1975), or the Balloon Analog Risk Task (Kóbor et al., 2021). People, however, also update their probability estimates (Behrens et al., 2007), which can attenuate these long-lasting effects of initial learning (Kóbor et al., 2021). Whether and how these potential primacy effects extend to risky decisions from experience is not known, however. Here, we assess two possible primacy effects in an experience-based risky-choice task, examining whether the initial probabilities (Exp. 1) or the initial relative values (Exp. 2) have long-lasting effects on choice.

When several options are learned within the same context, the range of possible outcomes can affect choice (e.g., Ludvig et al., 2014b; Soukupova et al., 2024). In the example in Figure 1, this range is 0 to 80, making 0 the worst outcome and 80 the best outcome in that context. Many studies have found that the best and worst outcomes in a context are overweighted: People are more risk-averse for a risky option that contains the worst possible outcome than for a risky option that contains the best possible outcome in the context. This bias is often referred to as the extreme-outcome effect (Konstantinidis et al., 2018; Ludvig et al., 2014b; see review by Madan et al., 2019). Moreover, the other outcomes in a context can alter risky choice (Madan et al., 2021). For example, given a choice between a risky option that yields 10 or 30 and a fixed option that yields 20, people are more risk-averse if the other outcomes in the context are higher (making 10 a low extreme) than if the other outcomes are lower (making 30 a high extreme). Thus, risk preference is affected by the relative value of the outcomes provided by the risky option. This learned relative value of an outcome is determined primarily by the other outcomes present in the encoding context—that is, the context in which a risky option is first experienced (Madan et al., 2021; Palminteri & Lebreton, 2021). This finding suggests that relative value may show a primacy effect in which the initial experiences are more influential than later experiences.

The present research thus systematically tested for potential primacy effects in risky decisions from experience. The first pair of experiments examined whether there was a primacy effect in these risky decisions due to the order of exposure to outcome probabilities. Would early exposure to better or worse outcome probabilities produce a long-lasting impact on choice? The second experiment examined whether there was a primacy effect due to the range of outcomes initially experienced. In addition to examining the impact on overall risk preferences for both gains and losses, the experiments also tested whether the initial conditions when learning about an option have a lasting and disproportionate effect on the extreme-outcome effect in risky choice (Ludvig et al., 2014b).

All experiments included three groups, two that received different orders of the manipulation, and one control group that received a neutral condition throughout. Experiments 1a and 1b varied the order of exposure to outcome probabilities for the risky options. For each risky option, the probability of getting the better of two outcomes was 50% across the entire session, but for one group it was higher (80%) in the first block and lower (20%) in the third block, whereas for another group this order was reversed (see Figure 1B). Experiment 2 kept the outcome probabilities for the two target risky options constant at 50%, but the relative values of those outcomes were manipulated by adding a third risky option (a wildcard) that either did or did not change the range of experienced outcomes (Table 1). Extreme wildcards, which gave better and worse outcomes than the target choices, occurred early in the session, later in the session, or not at all. In all experiments, the effect of order of exposure to the manipulations, and the presence of primacy effects, was assessed by comparing choices on later blocks during which all outcome probabilities were equal (Experiments 1a and 1b) or all wildcards were absent (Experiment 2).

**Table 1.** Summary of Experiment 2 Design. Points Provided by the Wildcard Door for Each Group During the Learning Blocks of Experiment 2.

Group	No extreme		Extreme first		Extreme last	
Blocks 1–3	45	55	5	95	45	55
Blocks 4–6	45	55	45	55	5	95

*Note.* All outcomes for risky target doors and for wildcard doors had a 50% chance throughout. No wildcard doors were presented for any group during block 7.

## Experiments 1a and 1b: Initial Experience With Different Outcome Probabilities

This set of two experiments varied the outcome probabilities for the risky options across blocks of the experiment, making the risky options objectively better than the fixed options during some blocks and objectively worse than the fixed options on other blocks. Across blocks, the probabilities were balanced such that after the third block, the risky and fixed options had been scheduled to provide the same overall number of points. On three subsequent blocks (Blocks 4–6), both outcomes were equiprobable for all risky options. The primary research question was whether choice on the last two blocks would show evidence of a primacy effect in which the initial experience carried more weight.

The testbed for these questions was an experience-based risky-choice procedure in which people choose between pairs of options, represented as doors (see Figure 1A; Ludvig et al., 2014b; Madan et al., 2014). Two of the options provided smaller numbers of points (low value), and two options provided higher numbers of points (high value). Within each value level, one option was fixed (i.e., gave the same number of points every time), and the other option was risky (sometimes gave more than the fixed option and sometimes less). When high- and low-value options are mixed within a session, people typically show an extreme-outcome effect whereby they make more risky choices for high-value than for low-value options, and they tend to overweight the best and worst outcomes in tests of memory (see Madan et al., 2019). Thus, in addition to assessing the effects of early experience on overall levels of risky choice, we also tested whether variations in win probability would alter this extreme-outcome effect.

### Method

#### Participants

Introductory psychology students at the University of Alberta (Experiment 1a: *N* = 192; 115 female; *M*_age_ = 19.7, *SD* = 3.2; Experiment 1b: *N* = 190; 136 female; *M*_age_ = 19.3, *SD* = 2.7) participated for course credit, as well as the opportunity to win a bonus of up to $5 CAD based on their performance in the study. Participants gave written informed consent, and the research was approved by the University of Alberta Research Ethics Board.

#### General Procedure

Participants were scheduled in groups of up to 15 and were given basic instructions in a large room. They were then assigned to small individual rooms where they played a computer-based task in which they were told to try to earn as many points as possible. As illustrated in Figure 1, participants were presented with pictures of one or two doors, and they indicated their choice by clicking on a door. Choices were immediately followed by feedback for 1.2 s, which showed the number of points won along with a cartoon “pot of gold” graphic. The total accumulated points were continuously displayed at the bottom of the screen, and immediate feedback was only supplied for the option chosen on each trial. An inter-trial interval that varied randomly from 1 to 2 s separated each trial.

As in several previous studies, there were four options, each represented by an image of a door (e.g., Ludvig & Spetch, 2011; Ludvig et al., 2014a, 2014b; Madan et al., 2014). Two doors always gave a fixed number of points: 20 for a fixed low-value (FL) door and 60 for a fixed high-value (FH) door. Two other doors were risky and gave one of two possible outcomes. The risky low-value (RL) door gave 0 or 40 points, whereas the risky high-value (RH) door gave 40 or 80 points. For these risky doors, the probability of receiving each outcome depended upon trial block, group assignment, and experiment (see Figure 1B).

Sessions were organized into six blocks of 80 trials, each separated by a brief break and a riddle. Each block included a mixture of three trial types. Decision trials involved choices between the RL and FL doors or between the RH and FH doors. On single-door trials, there was only one door presented, which had to be clicked to continue. These trials ensured that all doors were sometimes selected and that participants occasionally experienced all reward contingencies. Catch trials presented a choice between doors of different value (e.g., between FL and FH and between RL and RH). These trials assessed whether participants were paying attention, learned the outcomes, and were motivated to maximize points. Consistent with previous research, participants who chose the high-value door on fewer than 60% of catch trials during Blocks 5 and 6 were excluded from further analyses (Experiment 1a, *n* = 8; Experiment 1b, *n* = 7).

Each block had 40 single-door trials (10 for each door), 24 decision trials (12 for the low-value doors and 12 for the high-value doors), and 16 catch trials (2 for each combination of high-value vs. low-value door). Trial order was randomized within each block, and the total number of trials (480) and their distribution were constant across all participants. The specific door image associated with each option was counterbalanced across participants, and each door appeared equally often on both sides of the screen.

#### Group and Block Assignment

In Experiment 1a, the frequencies of the more extreme outcomes (0 and 80) for the risky options were manipulated together; in Experiment 1b, the frequencies of the better outcomes (40 and 80) were manipulated together. In both experiments, participants were randomly assigned to groups. The groups differed only in the probabilities assigned to the risky outcomes during Blocks 1 and 3. In Experiment 1a, participants in the baseline condition (Group EX 50-50) had an equal chance (50%) of getting either outcome for the risky options throughout the experiment. The two experimental groups had different probabilities of getting each outcome during the first and third blocks of trials. For participants in Group EX 80-20, there was an 80% chance of getting the extreme outcome for either risky option during the first block of trials. That is, for the RL door, there was an 80% chance of getting 0 points, and for the RH door, there was an 80% chance of getting 80 points. By contrast, participants in Group EX 20-80 had a 20% chance of getting the extreme outcomes (0 or 80 points) on the first block of trials. The probabilities were changed to 50% in the second block of trials and then were reversed in the third block (i.e., Group 1 had a 20% chance of an extreme outcome, while Group 2 had an 80% chance). For the last three blocks, the probabilities were again changed so that all groups had a 50% chance of getting either outcome.

In Experiment 1b, the procedure was identical except for the probability manipulations. The control group (BEST 50-50) had a 50% chance of either outcome on the risky options throughout. For Group BEST 80-20, the first block provided an 80% chance of getting the better outcome on both risky doors (i.e., 40 points for RL and 80 points for RH). For Group BEST 20-80, the first block provided only a 20% chance of getting the better outcomes on the risky doors. The outcome probabilities were then equated on Block 2 and reversed on Block 3 (i.e., 20% better outcomes for Group BEST 80-20 and 80% better outcomes for Group BEST 20-80). For the last three blocks, the probabilities were again changed so that all groups had a 50% chance of getting either outcome on risky choices.

#### Memory Self-Reports

After completing choice testing, participants were given two tests to assess their memory for the outcomes associated with each door. First, a recall test was given in which each door was presented in a randomly selected order, and the participant was asked to enter the number of points that first came to mind upon seeing that door. Second, a frequency-judgment test was given in which each door was shown again, in a new randomly selected order, along with the list of all outcomes in the task. The participant was asked to enter the percentage of time that door led to each outcome.

#### Data Analysis

All risky-choice proportions were calculated as the probability of choosing the risky option. ANOVAs were conducted using linear mixed-effects modeling fit by maximum likelihood to conduct a 3 × 2 mixed ANOVA with *p*-values and inverse Bayes factors (BF_10_) reported, as this approach necessitates fewer assumptions about distributional characteristics than conventional ANOVA. BF were approximated using the Bayesian information criterion, following the method described by Wagenmakers (2007; see also Jarosz & Wiley, 2014, for a practical guide to computing and interpreting BF). The risky-choice data were analyzed to address three questions: First, were participants sensitive to the probability manipulations in Blocks 1 and 3? Second, did participants in all groups show extreme-outcome effects? Third, was there evidence of a primacy effect as reflected in group differences in risky choice at the end of the session (Blocks 5 and 6) when all probabilities were equal?

The experimental task, data, and analysis code for this experiment are available on the Open Science Framework (OSF; https://osf.io/d85eu/).

### Results

#### Risky Choice

**Figure 2.**
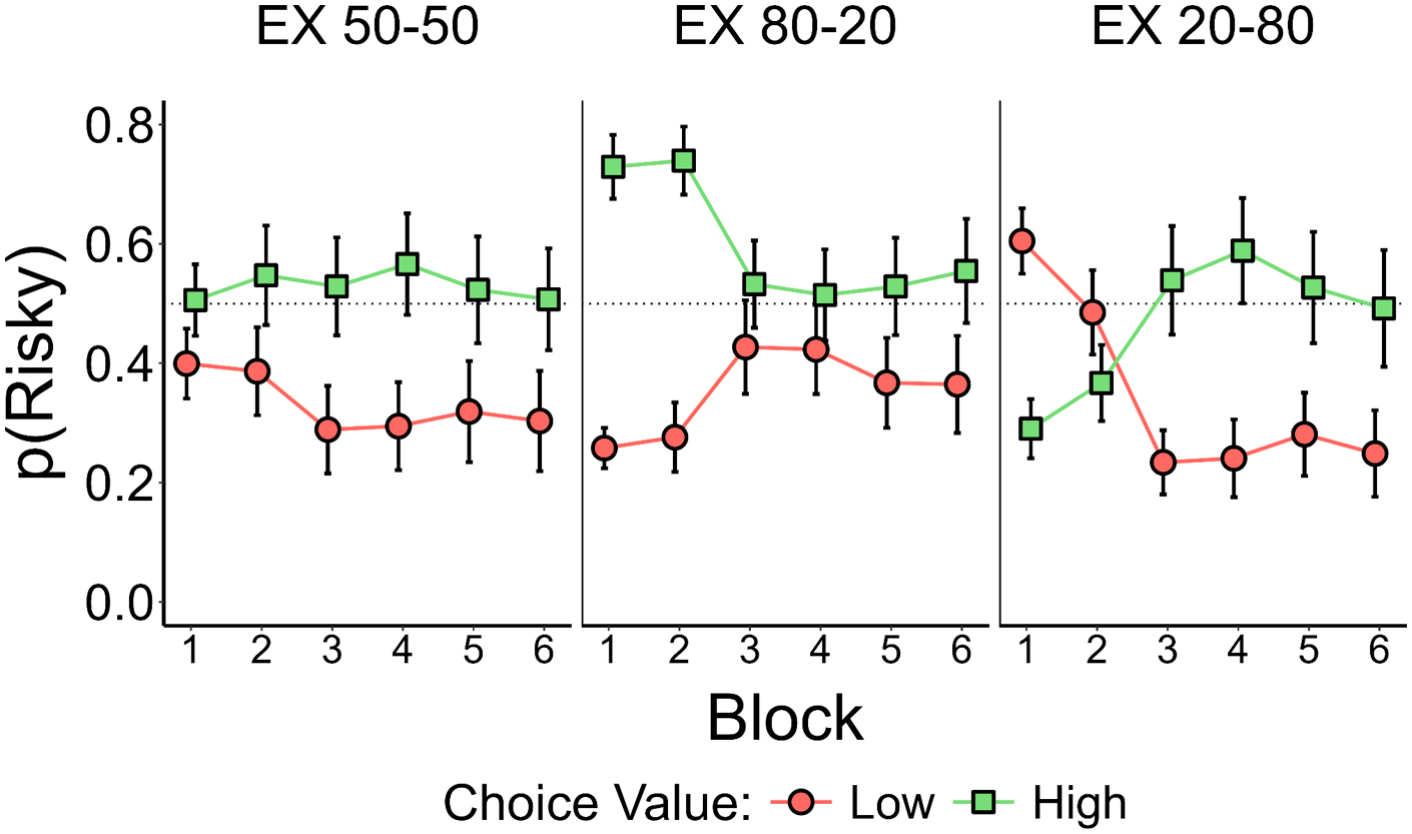
Proportion of risky choices on the low- and high-value choices across blocks for the control group (EX 50-50) and the two experimental groups of Experiment 1a. EX refers to extreme, and the numbers in the group names refer to the percent chance of receiving the extreme outcome during the first and third blocks. All other blocks provided a 50% chance of each outcome. Error bars are 95% confidence intervals.

Figure 2 shows the proportion of risky choices made by the three groups for low-value and high-value choices across the six blocks. As expected, on the first block, participants chose the risky option more often when the better outcome was delivered 80% of the time (i.e., for the high-value choices in EX 80-20 and the low-value choices in EX 20-80). These differences persisted in Block 2 when the probabilities shifted to being equal. In Block 3, when the probabilities were reversed, risky choice shifted as compared to Block 2, but only group EX 20-80 showed a full reversal.

By the end of the session, participants in all groups showed a robust extreme-outcome effect, choosing the risky option more often for high-value choices than for low-value choices, and there were no substantial differences between the groups. A two-way ANOVA, using linear mixed-effects modeling fit by maximum likelihood, with value as a within-subject factor and group as a between-subject factor showed a significant main effect of value, χ^2^(1) = 53.84, *p* < .001, BF_10_ > 150, confirming the extreme-outcome effect. There was no significant main effect of group, χ^2^(2) = 2.17, *p* = .34, BF_10_ < 0.01, and no significant interaction, χ^2^(2) = 1.19, *p* = .55, BF_10_ < 0.01.

**Figure 3.**
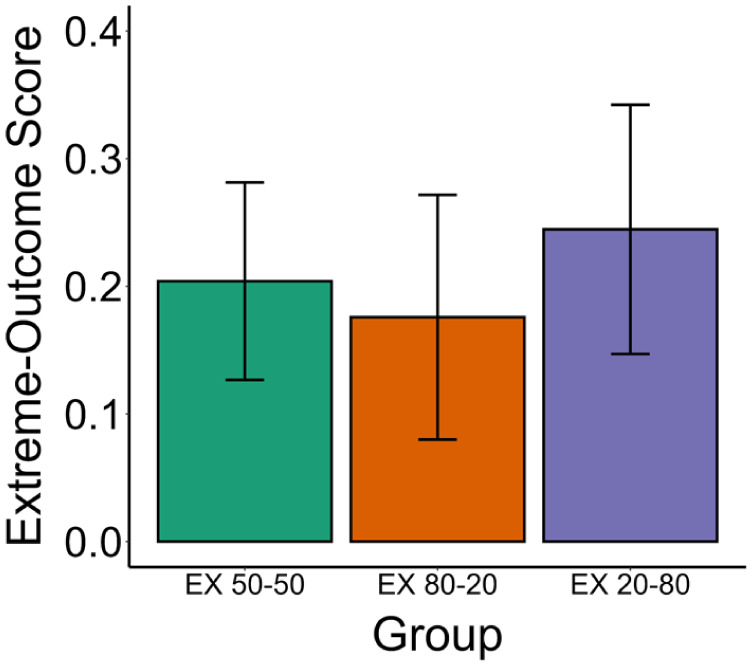
Extreme-outcome score (difference in the proportion of risky choices made for high-value choices versus low-value choices) for the three groups of Experiment 1a. Results are collapsed across Blocks 5 and 6, when all risky outcomes occurred with a 50% chance. EX is short for extreme, and the numbers in the group names refer to the percent chance of receiving the extreme outcome (80 points for high-value options and 0 points for low-value options) during the first and third blocks. Error bars are 95% confidence intervals.

Figure 3 shows this extreme-outcome effect (difference between high- and low-value risk preference) averaged over Blocks 5 and 6 for the three groups in Experiment 1a. In Group EX 50-50, people chose the high-value option 0.20 ± 0.077 (*M* ± MoE) more often than the low-value option, Cohen’s *d* = 0.69. In Group EX 80-20, people chose the high-value option 0.18 ± 0.10 more often than the low-value option, *d* = 0.46. Finally, in Group EX 20-80, people chose the high-value option 0.25 ± 0.1 more often than the low-value option, *d* = 0.64.

**Figure 4.**
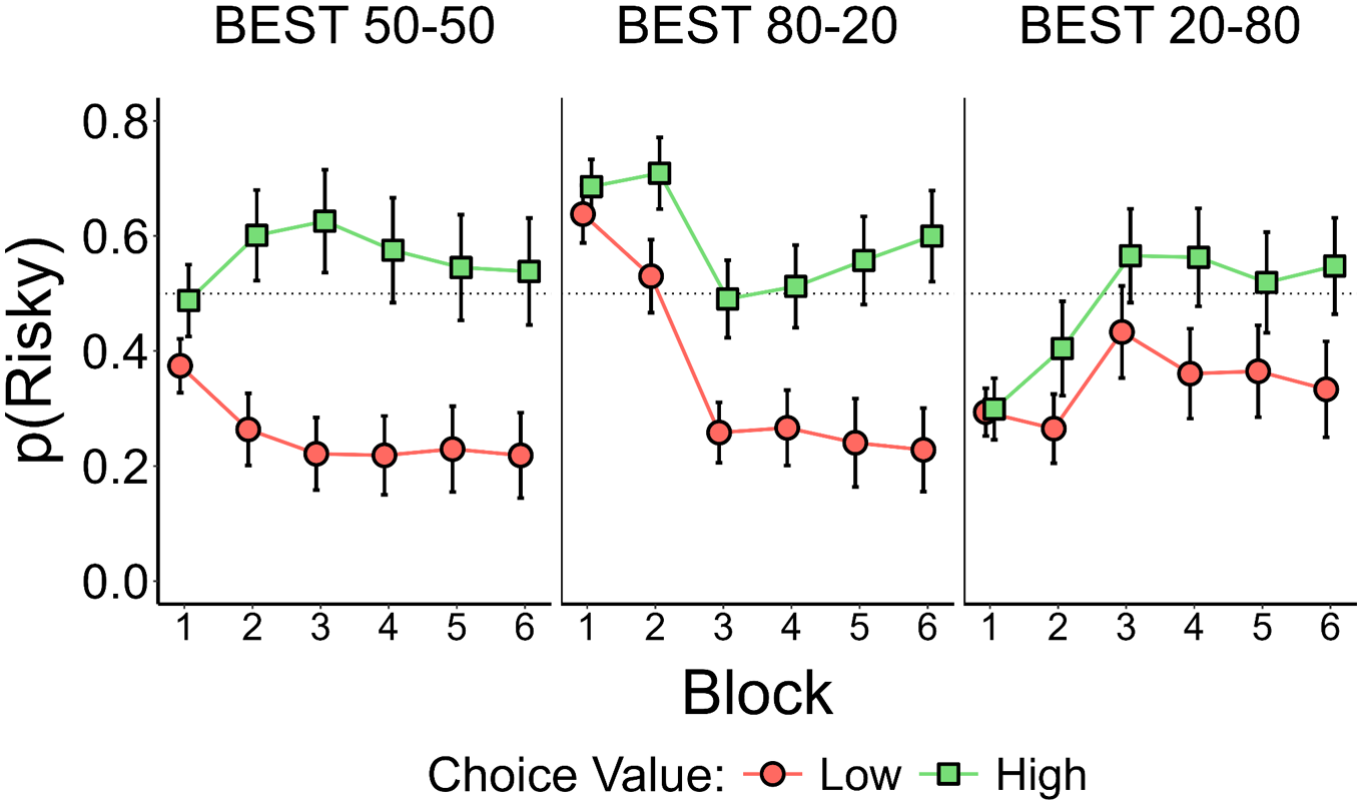
Mean proportion choice of the risky option on low- and high-value choices across blocks for the control group (BEST 50-50) and the two experimental groups of Experiment 1b. “BEST” refers to the higher of the two outcomes for the risky options, and the numbers in the group names refer to the percent chance of receiving this best outcome (i.e., 80 points for high-value options and 40 points for low-value options) during the first and third blocks. All other blocks provided a 50% chance of each outcome. Error bars are 95% confidence intervals.

In Experiment 1b, the three groups differed in the proportion of the better outcomes provided on the risky options. Figure 4 shows the proportion of risky choices made by the three groups on low-value and high-value choices across the six blocks. Again, as expected, in the first block participants consistently picked the option that led to an 80% chance of the better outcome (Group BEST 80-20) and avoided the options with only a 20% chance of the better outcome (Group BEST 20-80) as compared to the control group (Group BEST 50-50). These differences persisted in Block 2 when the probabilities shifted to being equal. In both experimental groups, risk preferences shifted on Block 3 as compared to Block 2 when the probabilities were reversed (see right two panels of Figure 4) and were then relatively consistent across the final three blocks (Blocks 4–6).

As in the previous experiment, there was a significant main effect of value, χ^2^(1) = 94.59, *p* < .001, BF_10_ > 150, but no significant main effect of group, χ^2^(2) = 1.65, *p* = .44, BF_10_ < 0.01. There was a significant interaction obtained between group and value, χ^2^(2) = 7.77, *p* = .021, BF_10_ = 0.13; however, in contrast to the *p*-value, the Bayes Factor showed moderate evidence against the presence of an interaction. The potential interaction in the frequentist statistic is likely due to the small difference between groups for low-value choices but not for high-value choices. Specifically, the BEST 20-80 group made slightly more risky selections on low-value choices than the other two groups. Planned comparisons assessing the interaction between group and value revealed that the difference between BEST 80-20 and BEST 50-50 groups did not significantly vary by value type, *t*(180) = 0.44, *p* = .66, *r* = .032. The difference between BEST 20-80 and BEST 50-50 groups, however, did vary significantly by value type, *t*(180) = −2.17, *p* = .031, *r* = .16. This mild interaction, however, is not in the direction predicted by a primacy effect.

**Figure 5.**
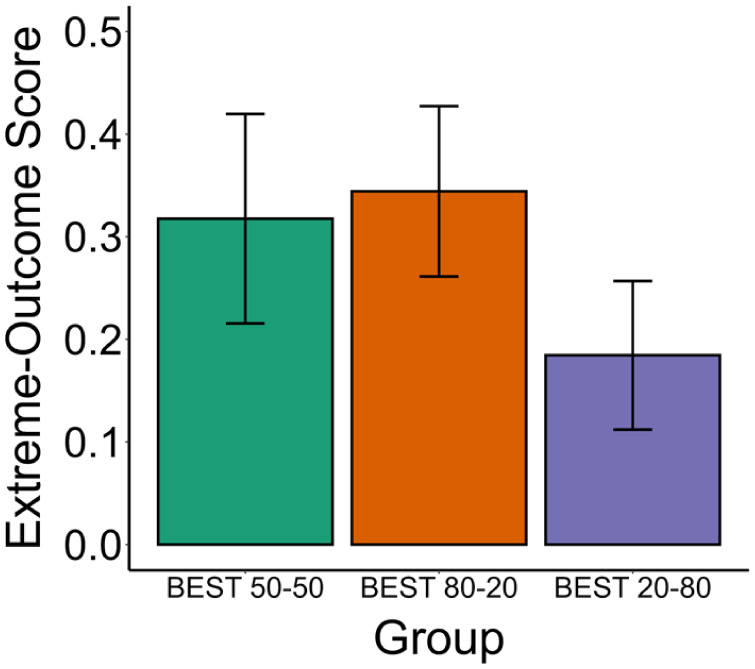
Mean extreme-outcome score (difference in the proportion of risky choices made for high-value choices versus low-value choices) for the three groups of Experiment 1b. Findings are collapsed across Blocks 5 and 6 for the control group (BEST 50-50) and the two experimental groups of Experiment 1b. “BEST” refers to the probability of the best risky outcome appearing, and the numbers in the group names refer to the percent chance of receiving the best outcome (80 points for high-value choices and 40 points for low-value choices) during the first and third blocks, respectively. All other blocks (including Blocks 5 and 6) provided a 50% chance of each outcome. Error bars are 95% confidence intervals.

Figure 5 shows the extreme-outcome score averaged over Blocks 5 and 6 for the three groups in Experiment 1b. In Group BEST 50-50, people chose the high-value option 0.32 ± 0.10 (*M* ± MoE) more often than the low-value option, Cohen’s *d* = 0.80. In Group BEST 80-20, people chose the high-value option 0.34 ± 0.08 more often than the low-value option, *d* = 1.06. Finally, in Group BEST 20-80, people chose the high-value option 0.18 ± 0.072 more often than the low-value option, *d* = 0.65.

#### Memory Tests

These results are presented in full in the supplementary materials (Supplemental Figures S1–S4) and were generally consistent with the choice results. In short, in all groups in both experiments, more people reported the extreme outcome (0 or 80) as the first outcome to come to mind (Supplemental Figures S1 and S2). In addition, almost all groups consistently judged the more extreme outcome as more likely, except for the BEST 20-80 group for the high-value risky options (Supplemental Figures S3 and S4). Full figures and inferential statistics are presented in the Supplemental Material.

### Discussion

In both experiments, after probabilities were re-balanced (i.e., in Blocks 5 and 6), there was no indication of any primacy effects in risky choice. That is, experiencing a lot of wins on a risky option in the first set of trials did not lead to a lasting increase in choice of that risky option later in that session. Participants in all groups in both studies, however, showed robust extreme-outcome effects, choosing the risky option more for high-value choices than for low-value choices (see Ludvig et al., 2014b). In addition, participants in all four experimental groups were sensitive to the outcome probabilities as indicated by the large group differences in the first block of trials. This finding differs from Weatherly and Brandt (2004) who found that people were not sensitive to payout percentages in a simulated slot machine. In addition to numerous procedural differences that could account for the discrepant results, the payout percentages in Weatherly and Brandt’s study varied over a smaller range (75%–95%) than in the present study (20%–80%).

## Experiment 2: Initial Experience With Different Relative Contexts

In Experiments 1a and 1b, there was no evidence of a primacy effect due to experience with different outcome probabilities. Experiment 2 assessed a different potential primacy effect: whether the relative values of outcomes in the first learning block affect later choice. The experiments used a similar design to Exp. 1 but added in a fifth “wildcard” option, which provided outcomes that were even more extreme than the ones experienced with the risky options. This wildcard option should reduce any extreme-outcome effect (e.g., see Figure 5) because the risky options no longer provide the best or worst outcomes in that context. This wildcard option was introduced either during initial learning trials (i.e., encoding) or on later blocks (after the contingencies have been learned). To equate experience with different numbers of options, the comparison conditions included a non-extreme wildcard that presented outcomes intermediate to the low-value and high-value outcomes. This non-extreme wildcard should not reduce the extreme-outcome effect because it does not change which outcomes are extreme.

There were three groups in the experiment, which differed in terms of their exposure to the extreme wildcard. Group Extreme-First received the extreme wildcard during initial learning (Blocks 1–3) and then the non-extreme wildcard on subsequent blocks (Blocks 4–6). Group Extreme-Last received the non-extreme wildcard during initial learning followed by the extreme wildcard on later blocks. Group No-Extreme was a control group that received non-extreme wildcards on all blocks. Risky choice was compared on a final block of test trials (Block 7) that was presented without feedback and without either wildcard option.

This experiment was pre-registered on the OSF (https://osf.io/yxcuh), and we made two opposing predictions based on results from Madan et al. (2021). Those experiments had people learn about the same target risky choice (i.e., +20 vs. 50/50 chance of +10 or +30) in two contexts—one where the other choices led to higher outcomes (e.g., +60 vs. 50/50 chance of +50 or +70) and one where the other choices led to lower outcomes (e.g., −20 vs. 50/50 chance of −10 or −30). Those same target choices were given in two visually distinct contexts that alternated across blocks. People chose differently pending the context: They were more risky when the target choice was in a context where all the other outcomes were lower, which aligns with the extreme-outcome rule, applied to that context. Crucially, on subsequent tests of the target risky choice in the other visual context, risk preferences reflected the relative values from the encoding context, not the test context. Based on this result, we proposed an *encoding hypothesis* for the current experiment, which predicts that the wildcard options that provide more extreme outcomes will affect risk preferences for the target choices and subsequent memories only when they are presented on early trials (i.e., during encoding). This hypothesis assumes that risk preferences develop early during learning and are relatively stable once learned. This prediction implies a smaller extreme-outcome effect for Group Extreme-First, but not for Group Extreme-Last, as compared to Group No-Extreme.

Alternatively, the context effect observed in the Madan et al. (2021) study may be dependent on the use of visually distinct choice stimuli (i.e., doors) in the two contexts. If this visual distinctiveness aspect is crucial for delineating contexts, then the presence of wildcard options might also alter choice even when presented after initial learning in the same context. This *whole-context hypothesis* would thus predict an effect of the extreme wildcard when presented either early or late in the sessions, leading to a smaller extreme outcome effect for both the Extreme-First and Extreme-Last groups as compared to the No-Extreme group.

### Method

#### Participants

Participants were recruited via Prolific Academic for an online experiment, using eligibility criteria of: (a) aged 18–65, (b) English as first language (self-reported), and (c) a Prolific Academic approval rating of over 90%. The target sample size was 336 (112 per group) to give 95% power to detect an effect size of Cohen’s *f* = 0.25 (corresponding to *r* = .24) with alpha set to .01. To allow for possible exclusions and unequal group distributions due to randomization, we recruited 390 total participants. As per the pre-registered exclusion criteria, 18 participants were excluded for failing to score above 60% correct on catch trials, leaving 372 total participants (221 female, 151 male) distributed among the three groups (Group Extreme-First: *N* = 141, Group Extreme-Last: *N* = 112, Group No-Extreme: *N* = 119). Participants were paid £4 for completing the task, plus an additional bonus of up to £5 based on performance (£1 per 4,000 points earned). The median task duration was under 30 min, and the average bonus was £3.77. The research was approved by the University of Alberta Research Ethics Board.

#### General Procedure

The general task was similar to the first experiment (see Figure 1), with pictures of doors serving as the choice stimuli and mouse clicks serving as the choice responses. Each choice was immediately followed by feedback about the points won for the chosen option, and the total accumulated points were continuously displayed at the bottom of the screen. After the feedback, a dot appeared in the middle of the screen and clicking on the dot initiated the next trial.

There were six different-colored doors in total. Four of these were the target options that were given throughout the experiment for all groups. Two of these doors gave fixed outcomes: Door FL always provided 30 points, and Door FH always provided 70 points. Risky doors provided two outcomes each with a 50% probability: Door RL gave 20 or 40, and Door RH gave 60 or 80 points. The remaining two doors were “Wildcard” choices that were given on certain blocks and differed across groups: a Wildcard Extreme door gave 5 or 95 points with a 50% chance of each, and a Wildcard Non-Extreme door gave 45 or 55 points with a 50% chance of each.

The session consisted of 7 trial blocks with 300 trials in total. Blocks 1 to 6 each had 44 trials, and Block 7 had 36 trials. Wildcards were presented only in Blocks 1 to 6, and the specific wildcard presented depended on Group. Group Extreme-First received the Wildcard Extreme door on Blocks 1 to 3 and the Wildcard Non-Extreme door on Blocks 4 to 6. Group Extreme-Last received the reverse order: Wildcard Non-Extreme on Blocks 1 to 3 and Wildcard Extreme on Blocks 4 to 6. Group No-Extreme served as the control group and received Wildcard Non-Extreme doors throughout. To equate for the stimulus change, the No-Extreme group received one door on Blocks 1 to 3 and then a different-colored door in Blocks 4 to 6, but the outcomes stayed the same.

For Blocks 1 to 6, each 44-trial block included a mixture of four types. There were 12 single-door trials, 2 for each of the 4 target doors and 4 for the wildcard door. These trials ensured that all doors were sometimes selected and that participants occasionally experienced all reward contingencies. There were eight catch trials that gave a choice between high- and low-value options: four presented a choice between FH and FL, and four provided a choice between RH and RL. These trials assessed whether participants were paying attention, learned the outcomes, and were motivated to maximize points. Participants who chose the high-value door on fewer than 60% of these trials were excluded from further analyses as per the pre-registration. There were 16 decision trials that involved choices between the fixed and risky doors. Eight of these were between FL and RL, and eight were between FH and RH. Finally, eight trials presented a choice between the wild-card door and a target door (two trials for each of the four target doors). The specific door image associated with each option was randomized across participants, and each door appeared equally often on both sides of the screen.

Block 7 served as a test block that was conducted without feedback and presented only the target doors. There were 12 catch trials and 24 decision trials. Participants were told that points would be awarded in the same way as before, but that they would no longer receive feedback following each choice.

#### Memory Tests

Choice trials were followed by two memory tests. On *First-Outcome-Reported Tests*, each door was presented individually (in a randomly selected order), and participants were asked to type the first outcome that came to mind for that door. On *Frequency-Judgment Tests*, participants again saw each door in a randomly selected order with two outcomes shown below each door. For the risky doors, these were the two outcomes that had occurred for that door. For the fixed doors, these were the outcomes that occurred for the two fixed doors. Participants were asked to type the percentage of time each of the two outcomes followed the displayed door. Responses needed to be a number in the range of 0 to 100.

#### Analysis

To remain consistent with the plan outlined in the pre-registration, the primary analysis was a one-way independent ANOVA that used generalized least squares (GLS), fit by maximum likelihood, to assess group differences (in contrast to the repeated-measures analyses central to Exp. 1a and 1b). As per the pre-registered analysis plan, we also conducted planned comparisons to specifically test the encoding hypothesis by comparing choices in Group Extreme-First to the other two groups. The primary measure for these comparisons was an extreme-outcome score calculated as the proportion of risky choices to high-value options minus the proportion of risky choices to low-value options. This score represents the strength of any extreme-outcome effect in choice, and the primary analyses were based on choices made on the seventh block (with no feedback). The prediction based on the encoding hypothesis is that the extreme-outcome effect will be lower in Group Extreme-First than in the other groups, which will not differ from each other (i.e., Extreme-First < Extreme-Last = No-Extreme). The prediction based on the whole-context hypothesis is that the effect will be lower in both extreme groups (i.e., Extreme-First = Extreme-Last < No-Extreme). In addition, two one-way GLS ANOVAs fit by maximum likelihood were separately run on the proportion of risky choices to the high-value options and the low-value options to assess any group differences specifically for the high or low extremes.

The experimental task, data, and analysis code for this experiment are available in OSF: https://osf.io/pnrwy/.

### Results

#### Risky Choice

**Figure 6.**
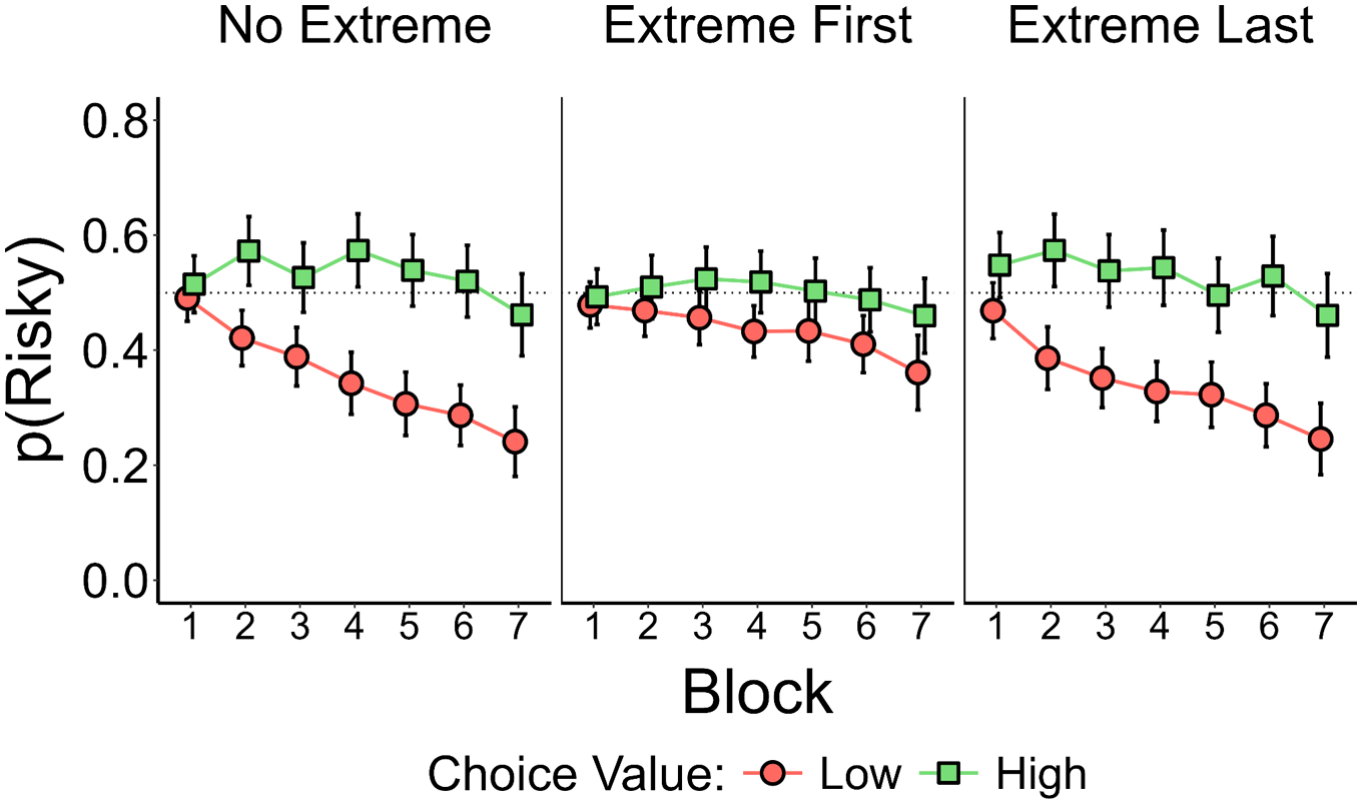
Proportion of the risky option selections for low-value and high-value choices across blocks for the control group and two experimental groups of Experiment 2. Group No-Extreme received non-extreme wildcards throughout Blocks 1 to 6. Group Extreme-First experienced an extreme wildcard on Blocks 1 to 3 and a non-extreme wildcard on Blocks 4 to 6. Group Extreme-Last received the non-extreme wildcard on the first three blocks and the extreme wildcard on Blocks 4 to 6. No wildcards occurred for any group on Block 7. Error bars are 95% confidence intervals.

**Figure 7.**
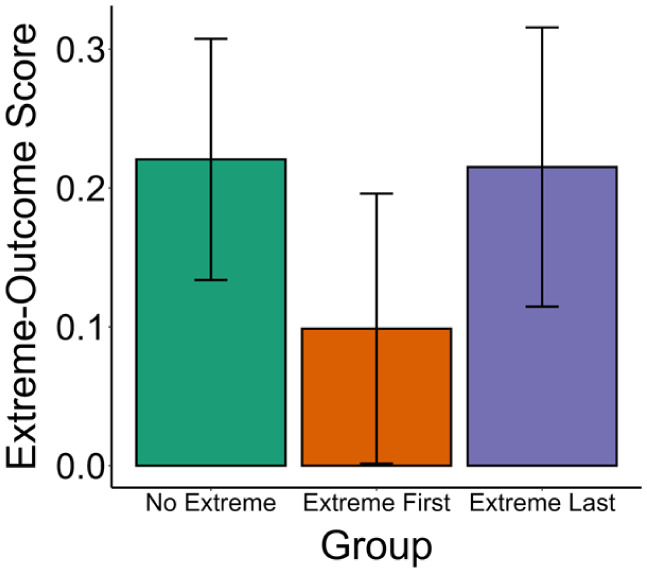
Mean extreme-outcome score (difference in the proportion of risky choices made for high-value choices vs. low-value choices) for the three groups of Experiment 2. Error bars are 95% confidence intervals.

Figure 6 shows the percentage of risky choices across blocks for the three groups on low- and high-value choices, and Figure 7 shows the extreme-outcome score on the test block for the three groups. Consistent with previous results, participants made more risky choices on high-value choices than on low-value choices on later blocks of trials, yielding an extreme-outcome score above 0. As would be expected from the encoding hypothesis, this extreme-outcome effect, however, was notably smaller in the Extreme-First group, which received extreme wildcards in the first block.

As pre-registered, we first analyzed risky choice separately for high- and low-value choices. A GLS ANOVA fit by maximum likelihood on high-value risky choices in Block 7 showed no significant group effect, χ^2^(2) = 0.001, *p* = .99, BF_10_ < 0.01, and none of the planned pairwise comparisons between the groups were significant. There was, however, a significant group effect on low-value choices, χ^2^(2) = 9.60, *p* = .008, BF_10_ = 0.33, and the planned comparisons showed that the Extreme-First group differed significantly from both the No-Extreme group *b* = −0.12, *t*(369) = −2.72, *p* = .007, *r* = .14, and the Extreme-Last group, *b* = −0.12, *t*(369) = −2.57, *p* = .011, *r* = .13. The No-Extreme group did not differ from the Extreme-Last group, *t*(228.3) = −0.11, *p* = .92, *d* = −0.01.

Figure 7 shows the extreme-outcome effect for Block 7 for the three groups in Experiment 2. In the No-Extreme group, people chose the high-value option 0.22 ± 0.087 more often than the low-value option, Cohen’s *d* = 0.46. In the Extreme-First group, people chose the high-value option 0.099 ± 0.097 more often than the low-value option, *d* = 0.17. Finally, in the Extreme-Last group, people chose the high-value option 0.22 ± 0.10 more often than the low-value option, *d* = 0.40.

A one-way GLS ANOVA fit by maximum likelihood on the extreme-outcome scores in Block 7 confirmed no significant main effect of group, χ^2^(2) = 4.31, *p* = .12, BF_10_ = 0.02. The pre-registered planned comparisons, however, supported the prediction that the Extreme-First group showed a smaller extreme-outcome effect than both the Extreme-Last group, *t*(369) = 1.71, *p* = .044, *r* = .089 and the No-Extreme group, *t*(369) = 1.82, *p* = .035, *r* = .09. A Welch’s two-sided *t*-test showed no significant difference in the extreme-outcome scores between the No-Extreme group and Extreme-Last group, *t*(222.18) = 0.08, *p* = .93, *d* = 0.01. These results provide support for the encoding hypothesis.

#### Memory Test: First Outcome Reported

**Figure 8.**
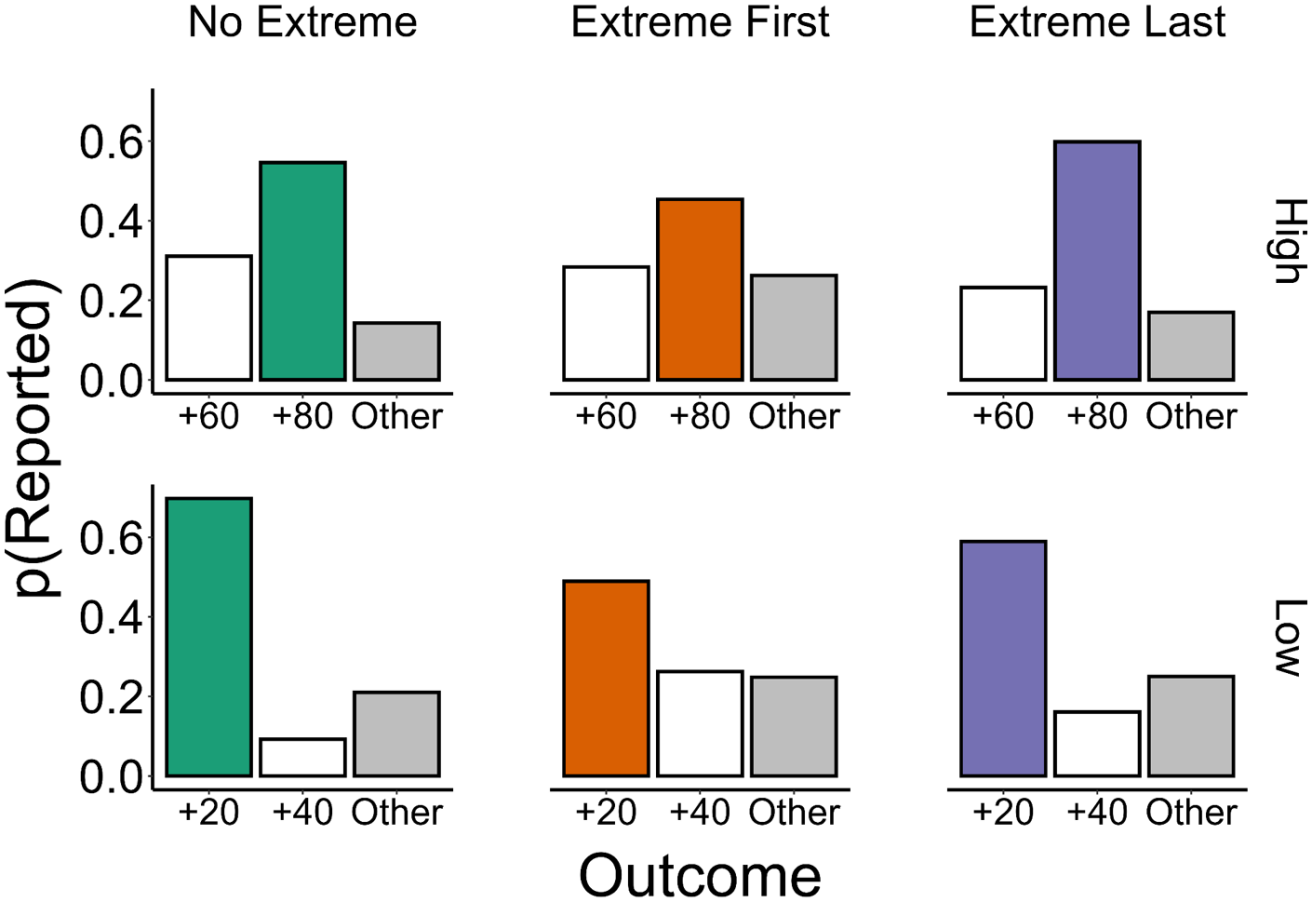
Results of the first-outcome-reported test for high-value risky options (top bars) and low-value risky options (bottom bars) for the three groups in Experiment 2. The values show the number of participants in each group who reported each outcome. Extreme outcomes (+20 and +80) are shown in color (with a different color for each group); non-extreme outcomes (+40 and +60) are shown in white. “Other” (shown in gray) is the proportion of participants who reported a number other than one of the outcomes experienced for that risky option.

Figure 8 shows the proportion of participants who reported the extreme outcome, non-extreme outcome, or another outcome for the high and low-value risky doors on the First-Outcome-Reported test of Experiment 2. As in Experiment 1 (see Supplemental Figures S1 and S2) and previous studies, people tended to report the extreme outcome (+20 or +80) over the non-extreme outcome (+40 or +60) for both risky options (high-value: χ^2^[1] = 27.68, *p* < .001, Cohen’s *w* = 0.34, and low-value: χ^2^[1] = 88.71, *p* < .001, *w* = 0.61). Using two separate 2 × 3 Pearson chi-squared tests of independence, comparisons for the high-value results were not significantly different, χ^2^(2) = 1.75, *p* = .42, ϕ_*c*_ = .09. In contrast, low-value results did differ across groups, χ^2^(2) = 8.48, *p* = .014, ϕ_*c*_ = .19. Post-hoc pairwise Fisher-Exact Tests, with a Holm-Bonferroni correction applied, found that reporting the extreme value was found to be dependent on group (*p* = .036) only when low-value options between the Extreme-First and No-Extreme groups are considered. This latter result fits with the encoding hypothesis.

#### Memory Test: Frequency Judgments

**Figure 9.**
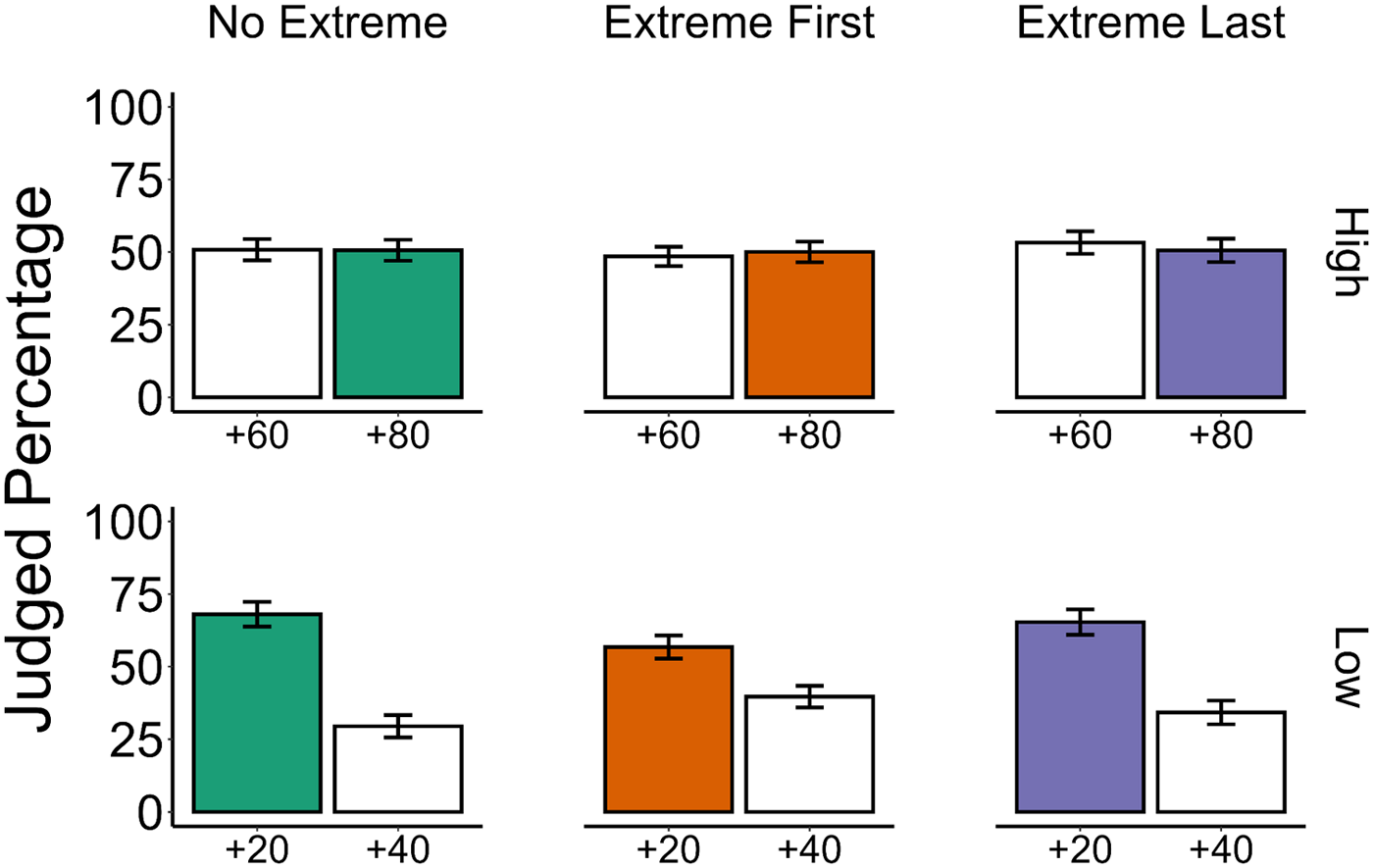
Results of the frequency-judgment test for high-value risky options (top bars) and low-value risky options (bottom bars) for the three groups in Experiment 2. The values show the average score reported when asked the percentage of time they remembered winning on that risky option. Extreme outcomes (+20 and +80) are shown in color (with a different color for each group); non-extreme outcomes (+40 and +60) are shown in white. Error bars are 95% confidence intervals.

Figure 9 shows the judged frequencies of the extreme and non-extreme outcomes for the high- and low-value risky doors on the Frequency-Judgment test of Experiment 2. Here, we conducted a 2 × 3 ANOVA using linear mixed-effects models fit by maximum likelihood to assess the effects of group and value. There was a main effect of value, χ^2^(1) = 57.18, *p* < .001, BF_10_ > 150, with greater judged frequencies for extreme outcomes for low-value options than high-value options. There was a main effect of group, χ^2^(2) = 9.36, *p* = .009, BF_10_ = 1.45, with lower judged frequencies for the extreme outcomes in the Extreme-First group than Extreme-Last group, *t*(368) = 2.22, *p* = .013, *r* = .12. The interaction was also significant, χ^2^(2) = 8.67, *p* = .013, BF_10_ = 0.103. Here, the magnitude of the value effect differed between the Extreme-First and No-Extreme groups, *t*(368) = 2.80, *p* = .005, *r* = .15. The magnitude of the value effect also differed between the Extreme-First and Extreme-Last groups, *t*(368) = 2.08, *p* = .038, *r* = .11. These results also provide support to the encoding hypothesis.

### Discussion

In this experiment, the range of values in the initial learning context impacted risky choice, especially for low-valued options. When the initial learning context contained a particularly low-valued option, then people were less risk-averse on the low-valued choice. This impact seems driven by the relative extremity of the worse option on the low-valued risky choice. Changing the range of values after that initial learning phase, by introducing an extreme wildcard later on, had no impact on risky choice (see Figures 6 and 7), nor on memory (see Figures 8 and 9). These results provide substantial support to the encoding hypothesis, whereby the range of values experienced during the period of initial learning is critical for establishing the pattern of risky choice (see also Madan et al., 2021).

## General Discussion

In this set of experiments, some aspects of initial learning impacted risky choice. A primacy effect was apparent with the relative values of outcomes, but was absent due to initial outcome probabilities. This novel primacy effect in risky choice builds on earlier work that has shown some impact of initial experience on real-world risky choices, as in stock-market decisions (Lejarraga et al., 2016) or in sports betting (Edson et al., 2021). Taken together, the results from this set of experiments suggest that learning about the relative value of an option follows different rules than learning about how likely the option is to pay off. Relative value appears to be learned early, based on the other outcomes in the initial learning context, and is reasonably stable thereafter. Outcome probability is also learned early, but it is updated after early learning, so that the tendency to choose the risky option reflects the overall probabilities rather than the probabilities experienced during initial learning.

Risky choices were affected by the presence of more extreme values provided by a wildcard option during initial learning, but not by the presence of more extreme values after learning had occurred. This finding complements the results of Madan et al. (2021) who found that the choice of a risky option was affected by the other outcomes present in the encoding context rather than the other outcomes present in the retrieval context. Their suggestion that relative value is established during encoding has implications for memory-based models of experience-based decision making (e.g., Shohamy & Daw, 2015; Weber & Johnson, 2013). In particular, the decision-by-sampling theory (Stewart et al., 2006) assumes that option values are compared with a small sample that is based on other values in the immediate context as well as values stored in long-term memory. The results of the present results together with those of Madan et al. (2021) suggest that experience-based risky choice is affected more by the other values in the encoding context than by the other values in the immediate choice context.

The primacy effect in relative value was much more pronounced on the low-value choices than on the high-value choices (see Figure 6). That differential impact may be because low-valued options are perceived as relative losses, compared to the reference point created by the range of outcomes (Kahneman & Tversky, 1979). People are often loss-averse and are also more attentive to losses (e.g., Yechiam & Hochman, 2013). This enhanced attention to losses may have made the lower outcome of the wildcard option more salient, thereby creating a larger impact on the lower-valued choices. In prior work, when queried about experienced options with equiprobable high and low outcomes, people tend to report the lower outcome as more frequent (Ludvig et al., 2015), corroborating this potential explanation. In addition, in all groups, the high-value choices hovered around 50%, suggesting that the main impact of extreme outcome on risky choice was driven by the low extreme in the first place. Thus, the inclusion of an even higher extreme through the wildcard did not impact risky choice, regardless of when it was introduced.

In contrast, the results of the first two experiments, which manipulated the probabilities of outcomes during an initial and a later block of trials, found no evidence of a primacy effect in learning about outcome probabilities. The two experiments in Experiment 1 manipulated the probabilities of obtaining the best or worst outcome for each risky option across blocks of trials. Group differences in risky choice on the first block showed that people were sensitive to the differences in outcome probabilities. After probabilities had been reversed and equated, however, risky choice showed no evidence of being more affected by the probabilities experienced during the initial learning than by those experienced later in the session. The reversal, however, likely created large prediction errors, which may have obscured any potential primacy effects, perhaps by creating a new context for subsequent decisions (Gershman et al., 2010; Rouhani et al., 2020). Nonetheless, all groups showed a robust extreme-outcome effect indicating that fluctuations in the probabilities of getting the best and worst outcomes do not interfere with the tendency to be more risk seeking for high-value options than low-value options, once the probabilities have been equated.

In the gambling literature, initial experiences with winning or losing have long been suggested to play a role in the propensity to continue gambling (Turner et al., 2006), although experimental evidence for a primacy effect has been mixed (Edson et al., 2021; Kassinove & Schare, 2001; Rune et al., 2012; Weatherly et al., 2004). Our results are consistent with those findings in suggesting that early strings of wins or losses are unlikely to have long-term effects on gambling propensity. On the other hand, the relative value of the outcomes when an option is first learned about may have more lasting effects. This suggests that the role of early experience with relative value in a gambling situation, such as whether a slot machine yields bigger wins or losses than others in the context, should be further explored.

## Supplemental Material

sj-docx-1-qjp-10.1177_17470218261432610 – Supplemental material for Effects of Initial Experiences on Risky Choice Supplemental material, sj-docx-1-qjp-10.1177_17470218261432610 for Effects of Initial Experiences on Risky Choice by Elliot A. Ludvig, Neil McMillan, Jeffrey M. Pisklak, Nick Simonsen, Alice Mason, Jason Long, Marcia L. Spetch and Christopher R. Madan in Quarterly Journal of Experimental Psychology
