# Supplementary material for: Effects of Initial Experiences on Risky Choice
Source: Q J Exp Psychol (Hove). 2026 Mar 4;79(8):2115–29. doi: 10.1177/17470218261432610 (PMC13400791; doi:10.1177/17470218261432610)
Supplement: sj-docx-1-qjp-10.1177_17470218261432610 – Supplemental material for Effects of Initial Experiences on Risky Choice [file sj-docx-1-qjp-10.1177_17470218261432610.docx]

Supplemental Materials for:

**Effects of initial experiences on risky choice**

Elliot A. Ludvig^1^, Neil McMillan^2,3^, Jeffrey M. Pisklak^2^, Nick Simonsen^4,6^, Alice Mason^5^,

Jason Long^2^, Marcia L. Spetch^2^, & Christopher R. Madan^6^

1. Department of Psychology, University of Warwick

2. Department of Psychology, University of Alberta

3. Medical School, University of Adelaide

4. Department of Management, Aarhus University

5. Department of Psychology, University of Bath

6. School of Psychology, University of Nottingham

Running head: Initial Experiences and Risky Choice

**Memory Results for Experiments 1a and 1b**

*First outcome reported.* Figures S1 and S2 show the proportion of participants who reported the extreme outcome, non-extreme outcome or another outcome for the high-value and low-value risky doors on the First-Outcome-Reported test of Experiments 1a and 1b. Consistently, across all groups, people tended to report the extreme outcome (0 or +80) over the non-extreme outcome (+40) for both risky options [Exp. 1a: high-value, *χ*^2^(1) = 40.75, *p* < .001, *w =* 0.50, and low-value, *χ*^2^(1) = 71.11, *p* < .001, *w =* 0.66; Exp. 1b: high-value, *χ*^2^(1) = 30.86, *p* < .001, *w* = 0.43, and low-value, *χ*^2^(1) = 85.71, *p* < .001, *w* = 0.71]. To assess whether this reporting of the extreme value was dependent upon group membership, two separate 2×3 Pearson chi-squared tests of independence were conducted for Experiments 1a and 1b’s high- and low-value results. For Experiment 1a, neither the high-value, *χ*^2^(2) = 0.81, *p* = .67, *φ_c_* = .07, nor the low-value results, *χ*^2^(2) = 3.04, *p* = .22, *φ_c_* = .14, yielded a statistically significant result for group. In Experiment 1b, for high-value options, reporting the extreme value was dependent on group, *χ*^2^(2) = 9.31, *p* = .01, *φ_c_* = .24, but for the low-value options, there was no significant difference, *χ*^2^(2) = 3.70, *p* = .16, *φ_c_* = .15. Post-hoc pairwise Fisher-Exact Tests, with a Holm-Bonferroni correction applied, found that the only significant difference was between the BEST 80-20 and BEST 20-80 groups (*p* = .022), where the tendency to report the higher number (80) was smaller in the BEST 20-80 group (see top right of Fig S2).


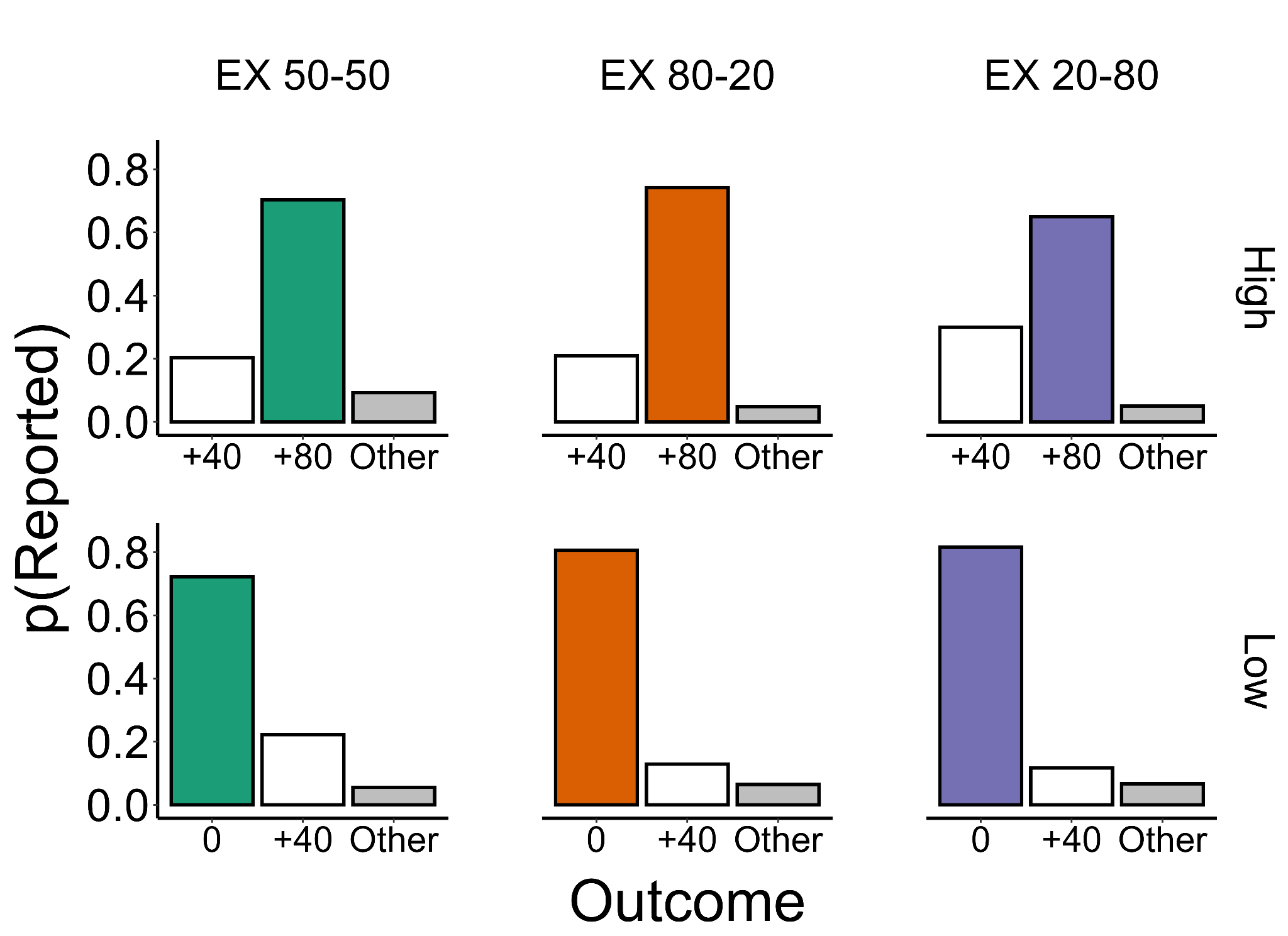


Figure S1. Results of the first-outcome-reported test for high-value risky options (top bars) and low-value risky options (bottom bars) for the three groups in Experiment 1a. The values show the proportion of participants in each group who reported each outcome. Extreme outcomes (0 and +80) are shown in color (with a different color for each group); non-extreme outcomes (+40 for both high and low value) are shown in white. “Other” (shown in gray) is the proportion of participants who reported a number other than one of the two outcomes experienced for that risky option.


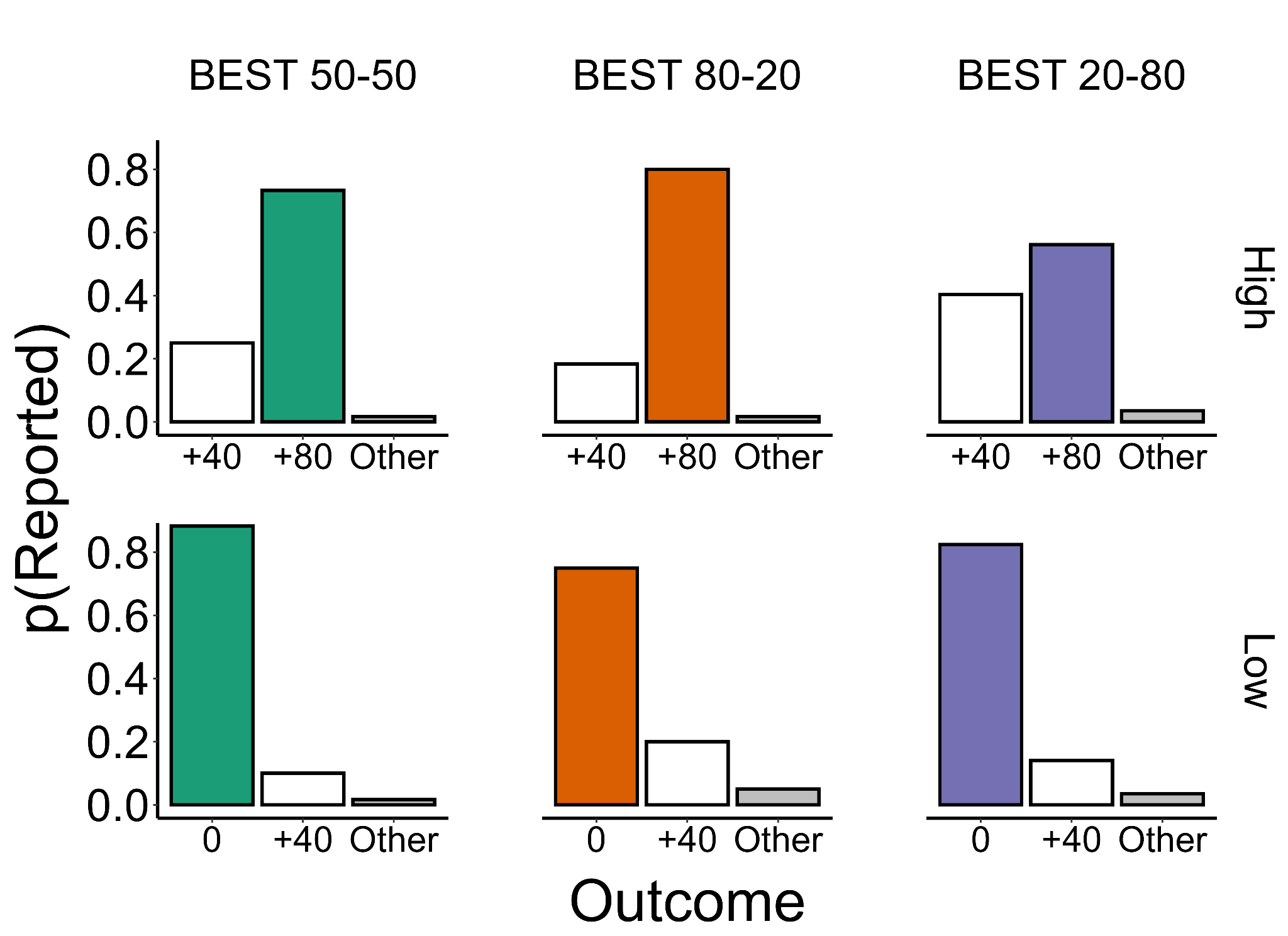


Figure S2. Results of the first-outcome-reported test for high-value risky options (top bars) and low-value risky options (bottom bars) for the three groups in Experiment 1b. The values show the proportion of participants in each group who reported each outcome. Extreme outcomes (0 and +80) are shown in color (with a different color for each group); non-extreme outcomes (+40 for both high and low value) are shown in white. “Other” (shown in gray) is the proportion of participants who reported a number other than one of the outcomes experienced for that risky option.

*Frequency judgments.* Figures S3 and S4 show the judged frequencies of the extreme and non-extreme outcomes, for the high- and low-value risky doors on the Frequency-Judgment tests of Experiments 1a and 1b. As expected, and in line with previous results, across almost all groups, people tended to judge the more extreme outcome as having occurred more frequently. This effect was much more pronounced in the low-value cases.

Here we conducted a 2×3 ANOVA using linear mixed-effects models fit by maximum likelihood to assess the effects of value and group, for each experiment. In Experiment 1a, there was a main effect of value, *χ*^2^(1) = 25.73, *p* < .001, 𝐵𝐹_10_ > 150, with higher judged frequencies for extreme outcomes in low- than high-value options, which agrees with previous studies. There was no main effect of group, *χ*^2^(2) = 0.12, *p* = .941, 𝐵𝐹_10_ < 0.01, nor an interaction, *χ*^2^(2) = 0.99, *p* = 0.61, 𝐵𝐹_10_ < 0.01. In Experiment 1b, the main effect of value was significant, *χ*^2^(1) = 67.85, *p* < .001, 𝐵𝐹_10_ > 150, again with higher judged frequencies for extreme outcomes in low- than high-value options. There were some evidence of a main effect of group, *χ*^2^(2) = 8.09, *p* = .018, 𝐵𝐹_10_ = 0.16, but there was again a contradiction between the *p*-value and Bayes Factor interpretation. The interaction was not significant, *χ*^2^(2) = 1.25, *p* = 0.54, 𝐵𝐹_10_ < 0.01.


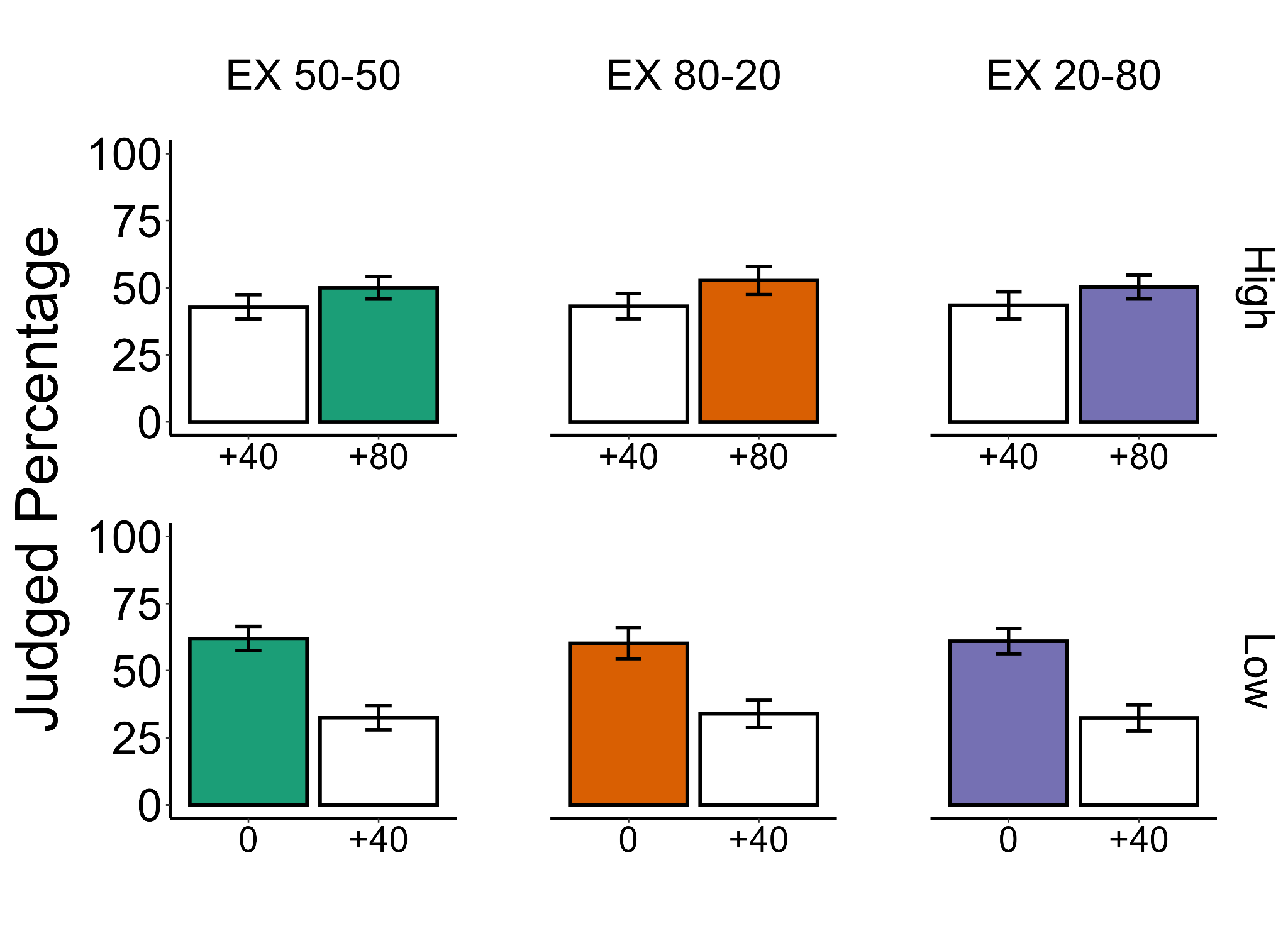


Figure S3. Results of the frequency-judgement test for high-value risky options (top bars) and low-value risky options (bottom bars) for the three groups in Experiment 1a. The values show the average score reported when asked the percentage of time they remembered winning on that risky option. Extreme outcomes (0 and +80) are shown in color (with a different color for each group); non-extreme outcomes (+40 for both high and low value) are shown in white. Error bars are 95% confidence intervals.


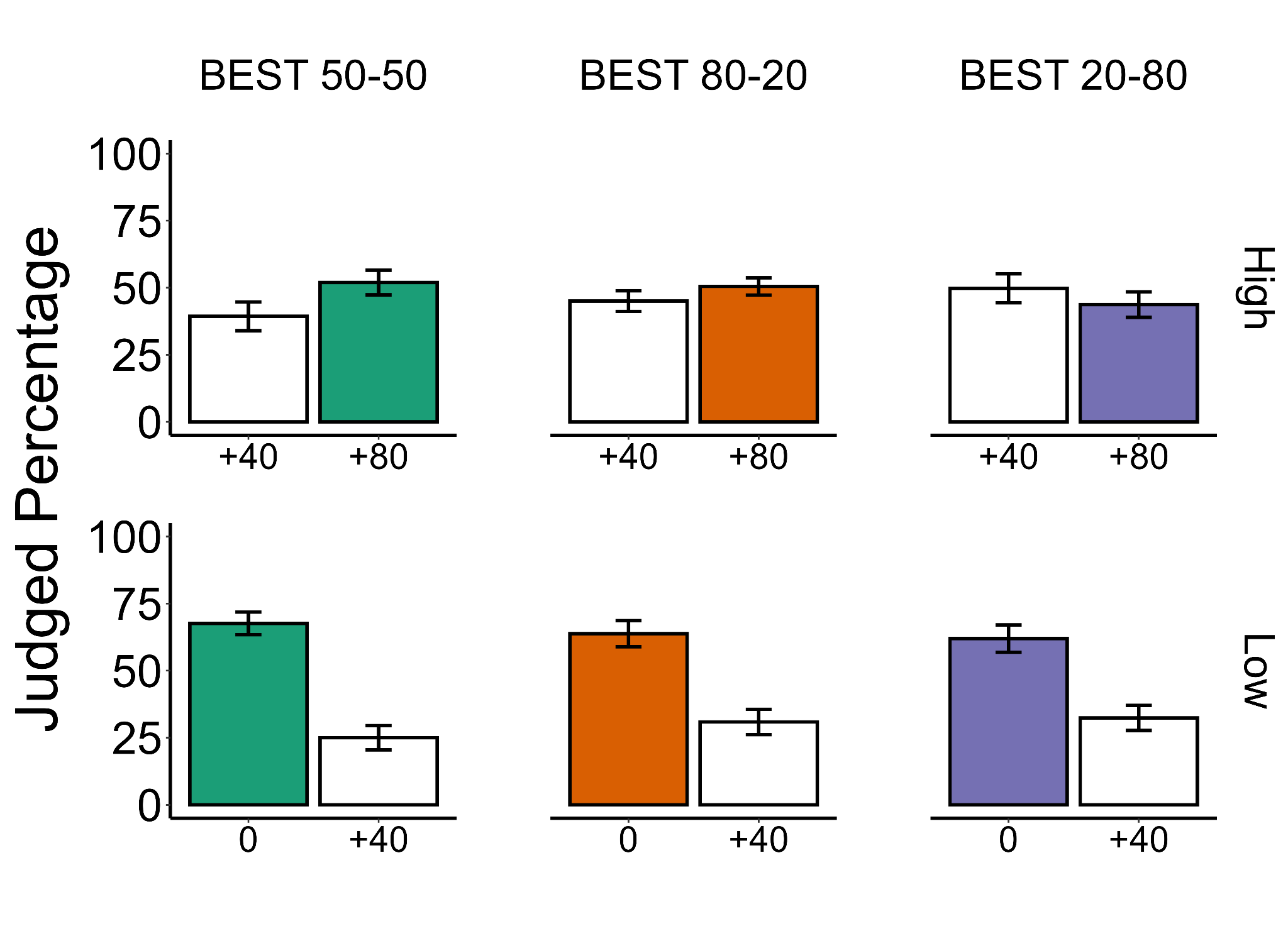


Figure S4. Results of the frequency-judgement test for high-value risky options (top bars) and low-value risky options (bottom bars) for the three groups in Experiment 1b. The values show the average score reported when asked the percentage of time they remembered winning on that risky option. Extreme outcomes (0 and +80) are shown in color (with a different color for each group); non-extreme outcomes (+40 for both high and low value) are shown in white. Error bars are 95% confidence intervals.
